# Performance enhancement of 6g millimeter wave links via RIS beamforming and CDL-based channel modeling

**DOI:** 10.1371/journal.pone.0358851

**Published:** 2026-09-24

**Authors:** KCT Swamy, Suman Turpati, Sivasubramanyam Medasani, Tathababu Addepalli, Bathula Ravi Chandra, Manish Sharma, Sameena Pathan

**Affiliations:** 1 GNSS & Communication Research Lab, Department of ECE, G. Pullaiah College of Engineering and Technology, Kurnool, India; 2 Department of Electronics and Communication Engineering, Dr K.V Subba Reddy Institute of Technology, Kurnool, India; 3 Department of CSE, K. S. School of Engineering and Management, Bengaluru, India; 4 Department of ECE, Aditya University, Surampalem, India; 5 Department of Electrical, Electronics and Communication Engineering, Galgotias University, Greater Noida, UP, India; 6 Manipal Institute of Technology, Manipal Academy of Higher Education, Manipal, India; Beijing Technology and Business University, CHINA

## Abstract

Communication using millimeter waves offer large bandwidth, become one of the key components of future sixth-generation wireless (6G) network. However, high levels of path loss and blockage limit the reliability of these types of links and communication, especially in non-line-of-sight (NLOS) scenarios. Recently, a novel technology known as reconfigurable intelligent surfaces (RIS) has been developed to help reconfigure the wireless propagation environment through programmable electromagnetic reflection. This paper discusses how using realistic cluster-delayed-line (CDL) channel modelling can improve the performance of RIS-assisted millimeter-wave communication links. A cascaded base station (BS)-RIS-user equipment (UE) system using a propagation channel that has been modelled according to mobile radio channel characteristics from the 3GPP Technical Report (38.901) was simulated at a frequency of 28 GHz in order to study the multipath clustering, angular dispersion and delay spread effects that take place when propagating signals using a wireless or radio channel. An iterative RIS phase optimization algorithm was developed for maximizing the coherent signal power obtained by combining all of the reflected signals based on the available directional antennas operated by RIS hardware. We present performance evaluations of the RIS-assisted channel compared to performance evaluations of traditional direct based channel configurations of identical communications facilities based on analysis of communications efficiency, signal-to-noise ratio, coverage probability, energy efficiency and accuracy of localization. The results show that RIS-assisted beamforming greatly improves communication performance from direct established links. For instance, when using 200 RIS elements, spectral efficiency of communication increases from approximately 2 bps/Hz to greater than 8 bps/Hz and the SNR at the receiver will have improved by almost 40 dB when using RIS technology compared to direct-established links.

## 1. Introduction

The rapid development of data-intensive applications, intelligent transportation systems, immersive multimedia services, and autonomous networks is hastening the evolution of wireless communication toward sixth-generation (6G) networks. Future wireless systems are expected to support extremely high data rates, ultra-low latency, massive device connectivity, and intelligent service provisioning. Millimeter-wave (mmWave) frequency bands have emerged as a key enabling technology for meeting these requirements, since they provide large available bandwidth and the ability to accommodate multi-gigabit communication links. MmWave signals, however, are faced with severe propagation challenges such as high path loss, spatial non-diffraction and blockage. These propagation constraints greatly reduce communication reliability, especially in dense urban and NLOS scenarios [1,2]. RIS has emerged recently to be a candidate solution that can reconfigure the wireless propagation environment in order to mitigate these problems. RIS generally consists of a large number of passive reflecting elements and its electromagnetic response can be dynamically changed by transmitting the signal through programmable phase shifts.

The RIS can modify the phase response of reflected signals to redirect the signal’s reflection and thus create better propagation paths and improve the strength of the signal received, especially in areas where blockage is expected. RIS has several advantages over traditional relay systems, such as lowered power consumption, simplified hardware requirements, and higher levels of energy efficiency, which makes them an attractive option for developing environmentally friendly communication system futures within the framework of 6G technologies [3,4]. Research into the RIS-augmented wireless communication systems is still ongoing; however, initial investigations have validated many of the fundamental principles of wireless communications as per intelligent reflecting surfaces (IRS) and indicated their potential for improving signal coverage and spectral efficiency [1]. Future studies will introduce an array of optimal designs for RIS-assisted beamforming to maximize throughput while minimizing overall energy consumption in multi-antenna communication environments [5,6]. Moreover, there has been an exploration of RIS-based signal processing procedures designed specifically to support secure communications, multi-user networks, and integrate for location/reason sensing applications [7–9]. Experimental studies have also been conducted to characterize the propagation behavior of RIS-assisted channels and develop path-loss models for practical deployments [10].

Further, many existing studies depend on simplified channel models that do not fully capture the complex multipath features observed in realistic wireless environments. Accurate channel modeling is vital for evaluating the performance of RIS-assisted systems in practical propagation circumstances. The cluster delay line (CDL) channel models standardized by the 3^rd^ Generation Partnership Project (3GPP) provide a realistic illustration of wireless propagation by integrating multipath clustering, angular dispersion, delay spread, and spatial correlation effects [11]. Although substantial advances have been made in RIS-assisted wireless communications, the majority of existing research literature adopts simplified geometric and/or statistical channel models that fail to adequately capture the clustered multipath propagation features of practical mmWave environments. A good performance of RIS relies heavily on the channel structure, and thus realistic channel modeling is a must for system-level evaluation. The 3GPP TR 38.901 Cluster Delay Line (CDL) channel models represent a standardized framework which includes multipath clustering, angular dispersion, delay spread, Doppler effects and spatial correlation. But the combination of CDL-based channel modeling and optimization of beamforming with RIS is not well studied. However, few studies have examined the performance of RIS-assisted communications under realistic CDL propagation scenarios, taking into account the practical optimization strategies for beamforming. The present study is an effort to fill this research gap.

Therefore, the main objectives of this paper are:

- To develop a framework for BS-RIS-UE cascaded channels with standardized CDL channel modeling that can capture multipath clustering, delay spread and angle dispersion in a mmWave environment.
- To design very efficient iterative phase alignment algorithm that maximizes the effective gain of the cascaded channel while providing low computational complexity, and practicable to implement on large arrays of RIS with wide-band OFDM.
- To evaluate the performance of RIS assisted communications system using metrics such as spectral efficiency, signal to noise ratio (SNR), coverage probability, energy efficiency & accuracy of positioning.
- To investigate the impact of RIS design parameters and propagation conditions in order to derive practical design guidelines for RIS-assisted communication in future 6G wireless networks.

The results provide insights into RIS deployment and highlight its potential to enable robust and sustainable intelligent radio environments for future 6G networks.

## 2. Related work

### 2.1 Overview of 6G technologies and enabling communication paradigms

The sixth generation (6G) wireless networks will deliver Ultra-high Data Rate, Ultra-low Latency, Massive Connectivity, High Reliability, and Precise Positioning capabilities to enable novel applications including Autonomous transportation, Industrial automation, Extended Reality (XR), Remote healthcare, and Intelligent Digital Infrastructure [12–15]. The vision, outlined by the International Telecommunication Union (ITU) in the IMT-2030, is that future 6G systems will deliver peak data rates of up to 1 Tbps, latency of less than 1 millisecond, and a faster synchronization rate of up to the microsecond scale, with a significant improvement in spectral and energy efficiency [12–15]. Beyond that, due to the fact that 6G networks are anticipated to use the same wireless infrastructure for both communication and sensing and localization, as well, Integrated Sensing and Communication (ISAC) is expected to be a key technology of the 6G networks [16,17]. AI is also expected to be a key enabler in network automation, resource management, and optimizing the adaptive beamforming performance to enhance the overall system performance [18].

To address those high expectations, 6G networks will combine terrestrial and non-terrestrial communication infrastructure, such as satellites, unmanned aerial vehicles (UAVs) and high-altitude platforms, in order to provide seamless connectivity globally across urban, rural, maritime and remote areas [19]. Moreover, the future wireless systems will make use of other spectrum bands, namely millimeter-wave (mmWave), terahertz (THz) and visible light communication (VLC) bands for ultra-high-capacity communications [20–22]. But there are significant propagation loss, blockage and coverage restrictions in these high frequency bands. Thus, cutting-edge physical-layer methods like beamforming, ultra-dense networks, and Reconfigurable Intelligent Surfaces (RISs) have been proposed as effective means to achieve better signal coverage, higher spectral efficiency, and reliable communication in challenging propagation conditions [20,21].

### 2.2 RIS-assisted communications and CDL-based channel modeling

Reconfigurable Intelligent Surfaces (RISs) are an emerging technology of future wireless networks that can boost spectral efficiency and enhance coverage and energy efficiency. Many articles have examined the RIS-assisted beamforming and phase optimization methods in idealized channel assumptions [1–7]. Recently, the capabilities of localization and sensing using RIS have been studied as well [8,9].

Many of these studies show significant performance improvements, but they use simplified channel models which ignore realistic multipath propagation characteristics. To overcome this, 3GPP has standardized CDL channel models that are able to model clustered multipath propagation, angular spreads, delay dispersion, angular correlation and Doppler effects in mmWave environments [11]. The use of CDL channel models in RIS-assisted communication systems is still fairly limited, however.

The concept of beamforming and the related spectral-energy efficiency trade-offs and blockage-aware beamforming have also been studied in recent work for mmWave networks [23,24]. Moreover, the integration of terrestrial, aerial and satellite communication networks, using advanced RIS-based beamforming architectures, has been proposed in [25–28]. However, a complete framework of CDL-based channel modeling, RIS-assisted beamforming optimization and performance evaluation of the system under realistic propagation conditions is still lacking.

For this reason, a cascaded BS–RIS–UE channel system based on CDL is proposed, and iterative RIS phase optimization for realistic 6G mmWave communication channels is explored.

### 2.3 Enabling applications and services

The world of wireless, through 6G technology, will transform from a series of separate networks into a seamless, fully integrated network of physical and digital infrastructures and will support:

- Autonomous vehicles and robotic systems
- AR/VR/MR and immersive 3D communication
- Tactile Internet for real-time remote interaction
- Remote healthcare and telesurgery
- Industry 5.0 and digital twins
- Precision agriculture and environmental monitoring

The Tactile Internet which will provide ultra-low latency communications for the real-time control and interaction with physical and virtual environments and is being supported by the efforts of the IEEE [29].

## 3. RIS-assisted 6G mmWave communication system

In this section, we present the mathematical model for RIS-assisted 6G mmWave communication systems. The mathematical formulation includes an end-to-end path of the cascaded BS-RIS-UE channels, the representation of OFDM signals, and how to control the phase of the RIS elements to offer increased reliability in NLOS conditions [30].

### 3.1 System geometry and deployment scenario

**Fig 1:**
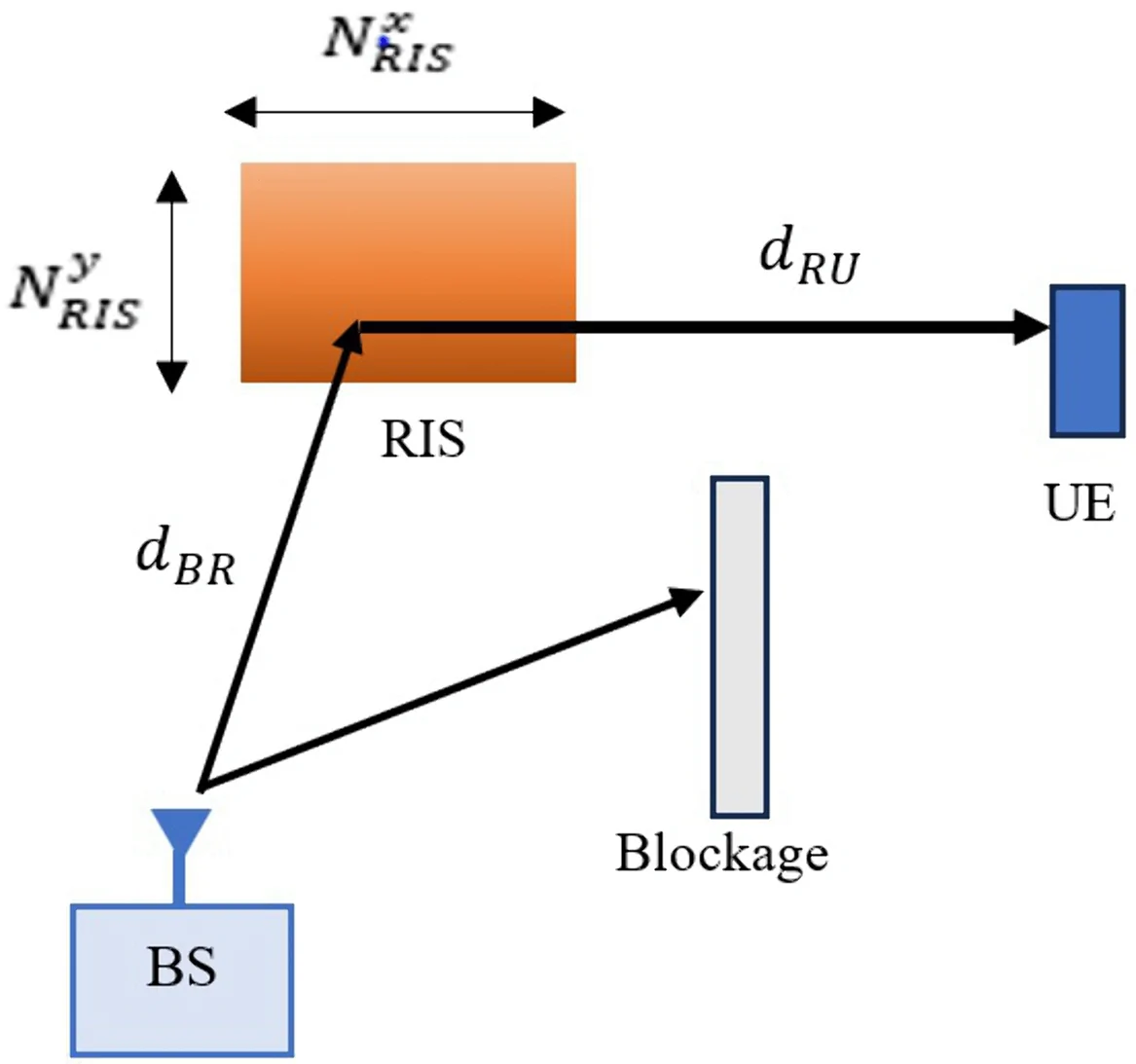
System geometry and cascaded channel paths (BS-RIS-UE).

Consider a downlink mmWave system operating at a carrier frequency of 28 GHz, a multi-antenna BS communicates with a single-antenna UE under NLOS blockage conditions (Fig 1).

A planar RIS is deployed to create a controllable reflected propagation path. Here, $N_{t}$ denote the number of transmit antennas of BS, N represents the number of RIS elements, $d_{BR}$ represents BS-RIS distance, $d_{RU}$ represents RIS-UE distance. The direct path is assumed severely attenuated due to blockage, making the RIS-assisted path dominant a typical scenario for mmWave urban deployments.

### 3.2 OFDM transmitted signal representation

The BS transmits a 6G-compatible OFDM waveform with subcarrier spacing Δf. The baseband signal is [31],


$$\begin{array}{c} x(t)=\sum_{k=\text{0}}^{K-\text{1}}X_{k}e^{j\text{2}\pi k\Delta ft} \end{array}$$ (1)

Where X_k_ denotes the 16-QAM symbol on subcarrier k.

For a multi-antenna transmitter,


$$\begin{array}{c} X_{k}=ws_{k} \end{array}$$ (2)

Where $s_{k}$ is the modulated symbol and w is the maximum ratio transmission (MRT) precoder. OFDM combined with beamforming is widely adopted for mmWave systems due to its robustness to frequency selectivity and spatial sparsity.

### 3.3 BS-RIS-UE channel modeling using CDL representation

The communication system comprises a base station (BS), an RIS having N programmable reflecting elements, and a user equipment (UE). To establish an additional controlled propagation path under blockage-dominated mmWave environment, the RIS is deployed. In Section 4, detailed formulation of the Cluster Delay Line (CDL) based channel is provided including the spatial correlation, delay spread, angular dispersion and Doppler effects. In this part, only the signal model from the system level is introduced.

### 3.4 RIS reflection matrix and phase control

The RIS applies programmable phase shifts to the impinging signal. The reflection matrix is [30]


$$\begin{array}{c} \Theta =\text{A diag}\left(e^{{j\emptyset }_{\text{1}}},e^{{j\emptyset }_{\text{2}}},\ldots \ldots \ldots \ldots e^{{j\emptyset }_{N}}\right) \end{array}$$ (3)

where A is the reflection amplitude and $\emptyset_{\text{N}}$ denotes the phase shift applied by the n^th^ RIS element.

Programmable phase control enables shaping of the electromagnetic wavefront to achieve constructive combining at the receiver.

### 3.5 Cascaded RIS channel formulation

The effective RIS-assisted channel is [30].


$$\begin{array}{c} {𝐡}_{eff}={𝐡}_{RU}^{T}{\Phi 𝐇}_{BR} \end{array}$$ (4)

The received signal on subcarrier k becomes

where $n_{k}\sim CN(\text{0},\sigma^{\text{2}})$.

This cascaded formulation is widely adopted for RIS-assisted links.

### 3.6 Optimal phase alignment

To maximize received signal power, RIS phase shifts are selected to align the reflected components coherently [31],


$$\begin{array}{c} \phi_{n}^{opt}=-\text{arg}(h_{RU,n}{\left({𝐇}_{BR}𝐰\right)}_{n}) \end{array}$$ (5)

This alignment enables constructive superposition of reflected waves at the UE. The extension to this optimal phase design of frequency-selective wideband CDL channels is discussed in Sec 5.

### 3.7 Signal-to-noise ratio (SNR)

The received SNR per subcarrier is


$$\begin{array}{c} \gamma =\frac{P_{t}{|{𝐡}_{RU}^{T}{\Phi 𝐇}_{\text{BR}}𝐰|}^{\text{2}}}{\sigma^{\text{2}}} \end{array}$$ (6)

Under ideal phase alignment, $\gamma \propto N^{\text{2}}$

demonstrating quadratic array gain, a fundamental advantage of RIS-assisted propagation.

### 3.8 Spectral efficiency

The achievable rate per subcarrier is


$$\begin{array}{c} R={\text{log}}_{\text{2}}(\text{1}+\gamma ) \end{array}$$ (7)

and total throughput is,


$$\begin{array}{c} R_{tot}=B{\text{log}}_{\text{2}}(\text{1}+\gamma ) \end{array}$$ (8)

RIS-assisted beamforming can significantly improve spectral efficiency in blockage-limited mmWave systems.

### 3.9 Energy efficiency

Energy Efficiency (EE) refers to the amount of energy that can be achieved by a specific energy system divided by the total amount of energy consumed by the system. In reality, RIS-assisted systems require power in the RIS controller, phase-control electronics, and signal-processing modules, as well as transmit power. So, the energy efficiency is given by,


$$\begin{array}{c} EE=\frac{R_{total}}{P_{tot}} \end{array}$$ (9)

where $R_{total}$ denotes the achievable system throughput and the total power consumption ($P_{tot}$) is


$$\begin{array}{c} P_{tot}=P_{t}+P_{RIS}+P_{ctrl}+P_{proc} \end{array}$$

Here, $P_{t}$ = BS transmit power, $P_{RIS}$ = power consumed by RIS reflecting elements, $P_{ctrl}$= RIS controller and phase-control circuitry power, $P_{proc}$ = baseband signal-processing and optimization overhead.

Consequently, $EE=\frac{B{\text{log}}_{\text{2}}(\text{1}+\gamma )}{P_{t}+P_{RIS}+P_{ctrl}+P_{proc}}$

where *B* represents the bandwidth of the system, and γ the received S/N ratio.

While RIS-assisted systems also create extra control and processing overhead, they do not consume much power compared to active relay systems, since no RF amplification chains are required for RIS elements to act as passive electromagnetic reflectors. It is therefore possible to significantly improve spectral efficiency of RIS-assisted communication systems with high energy efficiency.

Unlike active relays, which must be radio-frequency amplified, frequency converted and signal regenerated, RIS elements are almost passive reflecting elements. Both experimental and analytical studies have demonstrated that the energy consumption of practical RIS controllers is usually several orders of magnitude less than that of conventional relay stations, rendering RIS a highly energy-efficient solution for future wireless networks [2,3,26].

### 3.10 RIS-enhanced localization and sensing

In addition to communication enhancement, RIS can be used to effectively enhance the performance of user localization in wireless systems through controllable propagation paths to increase spatial diversity and angular resolution. In the considered scenario, localization is obtained from the propagation link between BS and RIS, and between RIS and UE, such as Angle-of-Arrival (AoA), Angle-of-Departure (AoD), and propagation delay measurements.

The reflected signals can be steered in the desired direction by suitably modifying the RIS phase shifts, leading to better angular discrimination at the receiver. These extra reflected paths created by the RIS, in turn, increase the number of observable propagation paths that can be used for positioning, resulting in greater geometric diversity. Thus, RIS channels can be used in blockage-dominated Non-Line-of-Sight (NLOS) environments to also deliver localization information. The localization performance is assessed with the Fisher Information Matrix (FIM) which measures the information that can be obtained about the user’s position. The FIM is defined as [32–36],


$$\begin{array}{c} 𝐉\left(\theta \right)=E\left[\left(\frac{\partial \text{ln}p\left(y|\theta \right)}{\partial \theta }\right){\left(\frac{\partial \text{ln}p\left(y|\theta \right)}{\partial \theta }\right)}^{T}\right] \end{array}$$ (10)

The likelihood function for the received observations p(y∣θ) is a function of the position parameters, θ.

The achievable localization accuracy is assessed by the Cramér–Rao Lower Bound (CRLB), which gives the theoretical lower bound on the variance of the estimation:


$$\begin{array}{c} \text{CRLB}={𝐉}^{-1}\left(\theta \right) \end{array}$$

The smaller the CRLB, the more accurate the positioning. Because of the enhancement of received signal strength, angular resolution, multipath diversity, with RIS, the corresponding FIM also improves, and the CRLB decreases, which leads to better localization performance.

The Root Mean Square Error (RMSE) of the estimated position is then chosen as a practical performance metric for localization and compared for various RIS sizes and propagation scenarios in the Results section.

## 4. CDL-based cascaded channel modeling

The 3GPP TR 38.901 CDL framework explicitly describes: multipath clustering, delay spread, angular spread, spatial correlation, Doppler spectra, frequency-selective fading. These properties are crucial for RIS-assisted millimeter-wave systems, where the propagation environment plays a key role in the beamforming performance.

### 4.1 Motivation and significance of CDL-based modeling

3GPP TR 38.901 presents an entirely new set of spatial channel models known as the Clustered Delay Line (CDL) channel models, allowing for a standard and geometry consistent representation of wireless propagation environments [11]. CDL channel models will provide a higher level of fidelity in the representation of spatial and temporal characteristics of wireless channels when compared to conventional statistical or simplistic geometric channel models. CDL models contain spatially clustered multipath components, angular spreads of arrival and departure in both azimuth and elevation, delay spread, Doppler shift, and spatial correlation across antenna arrays, all of which are important for advanced communication systems, such as mmWave communication systems and systems employing RIS to assist in wireless communication where signal propagation is primarily determined by directional RF scattering and reflection.

The CDL profiles represent the different propagation conditions for which they are designed to represent, based upon the cluster definitions characterizing the clustering of multiple reflections in an environment (including angles of arrival and departure and timing of arrival) defined by three parameters: i.e., the angular spread, delay spread, and multipath characteristics. CDL-D represents representative NLOS propagation characteristics, while CDL-E represents representative LOS propagation characteristics. Therefore, CDL profiles serve to accurately represent realistic wireless channels in complex propagation environments, particularly in high-frequency systems in which the presence of clustered multipath is significant on overall wireless system performance.

In RIS-assisted communication systems, CDL channel models are critical to look at how the intelligently controlled reflections being created by the RIS will interact with UE.

### 4.2 BS-RIS channel modeling

The BS–RIS channel is modeled as a multi-cluster MIMO channel:


$$\begin{array}{c} {𝐇}_{BR}\left(t,f\right)=\sum_{\text{c}=\text{1}}^{\text{C}}\sum_{\text{r}=\text{1}}^{{\text{R}}_{\text{c}}}\alpha_{c,r}{\text{e}}^{-j\text{2}\pi f\tau_{c,r}}{𝐚}_{\text{RIS}}\left(\theta_{c,r}^{\text{RIS}},\phi_{c,r}^{\text{RIS}}\right){𝐚}_{\text{BS}}^{𝐇}\left(\theta_{c,r}^{\text{BS}},\phi_{c,r}^{\text{BS}}\right) \end{array}$$ (11)

Where, C= number of scattering clusters, $R_{c}$= number of rays in cluster, $\alpha_{c,r}$ = complex path gain, $\tau_{c,r}$= delay, and θ, ϕ denote azimuth and elevation angles.

The CDL representation explicitly models clustered multipath propagation by multiple delay taps and angular clusters. The array response vectors of different angles of arrival and departure create naturally the spatial correlation, and the propagation delay (or delays) of the cluster creates the delay spread (or delays) $\tau_{c,r}$.

Therefore, CDL is a representative model of the mmWave propagation channel, which cannot be captured by the simplified narrowband channel model.

The array response vector for a uniform planar array (UPA) is


$$\begin{array}{c} 𝐚(\theta ,\phi )=\frac{\text{1}}{\sqrt{\text{N}}}{[{\text{e}}^{\text{j}\frac{2\pi }{\lambda }\text{k}\left(\theta ,\phi \right).{\text{r}}_{\text{1}}},...,{\text{e}}^{\text{j}\frac{2\pi }{\lambda }\text{k}\left(\theta ,\phi \right).{\text{r}}_{\text{N }}}]}^{\text{T}} \end{array}$$ (12)

Where k(θ,ϕ)is the wave vector and $r_{n}$denotes element positions. This formulation captures the spatial selectivity and angular sparsity typical of mmWave channels.

### 4.3 RIS-UE channel modeling

The RIS–UE link is modeled similarly:


$$\begin{array}{c} {𝐡}_{RU}\left(t,f\right)=\sum_{c=\text{1}}^{C^{1}}\sum_{r=\text{1}}^{R_{c}^{1}}\beta_{c,r}e_{c,r}^{-j\text{2}\pi f\tau^{1}}a_{RIS}\left(\theta_{c,r}^{1},\phi_{c,r}^{1}\right) \end{array}$$ (13)

Because of the low propagation distance and orientation reflective properties, RIS-UE channels normally have a sparse angular structure much like mmWave links.

### 4.4 Cascaded channel formulation

The effective RIS-assisted channel is the cascade:


$$\begin{array}{c} {𝐡}_{eff}\left(t,f\;\right)={𝐡}_{RU}^{T}\left(t,f\right){\Phi 𝐇}_{BR}(t,f\;) \end{array}$$ (14)

Substituting the CDL formulas we can determine,


$$\begin{array}{c} {𝐡}_{eff}=\sum_{c,r}\sum_{c^{1},r^{1}}\beta_{c^{1},r^{1}}\alpha_{c,r}e^{{-j\text{2}\pi f(\tau_{c,r}+\tau^{1}}_{c,r})}{𝐚}_{RIS}^{T}(\theta_{c,r}^{1}){\Phi 𝐚}_{RIS}(\theta_{c,r}^{RIS}){𝐚}_{BS}^{H}(\theta_{c,r}^{RIS}) \end{array}$$ (15)

This equation shows that RIS-assisted propagation is governed by double scattering, where each RIS element contributes to coherent combining of multipath components.

### 4.5 Path loss and large-scale fading

The cascaded path loss follows the ETSI RIS far-field model [1]:


$$\begin{array}{c} {PL}_{RIS}=PL(d_{BR})\times PL(d_{RU}) \end{array}$$ (16)

$$\begin{array}{c} PL\left(d\right)=C_{\text{0}}{\left(\frac{d}{d_{\text{0}}}\right)}^{-\alpha } \end{array}$$ (17)

where: α is the path-loss exponent, $C_{\text{0}}$is reference loss.

Thus, ${PL}_{RIS}\propto d_{BR}^{-\alpha }d_{RU}^{-\alpha }$. This multiplicative attenuation distinguishes RIS propagation from conventional single-hop channels.

### 4.6 Spatial correlation and angular resolution

RIS aperture size determines angular resolution:


$$\begin{array}{c} \Delta \theta \approx \frac{\lambda }{D} \end{array}$$ (18)

Where D is RIS aperture dimension. Larger RIS panels provide finer beam control and improved spatial selectivity, enabling enhanced beamforming and localization performance.

### 4.7 Near-field and far-field considerations

For large RIS apertures, the far-field assumption may break down. The Fraunhofer distance is [30],


$$\begin{array}{c} d_{F}=\frac{{\text{2}D}^{\text{2}}}{\lambda } \end{array}$$ (19)

When$d<d_{F}$, spherical wavefront modeling becomes necessary. Near-field RIS behavior is an emerging research area in 6G systems due to extra-large intelligent surfaces.

### 4.8 Frequency selectivity in OFDM systems

Delay spread from CDL clusters produces frequency selectivity across OFDM subcarriers:


$$\begin{array}{c} H_{k}=\sum_{l}h_{l}e^{-j\text{2}\pi k\Delta f\tau_{l}} \end{array}$$ (20)

RIS phase optimization may be narrowband (single phase profile), or wideband (sub band-adaptive control). Wideband optimization improves performance in frequency-selective channels. The corresponding iterative wideband phase optimization is presented in Sec. 5.

### 4.9 Doppler effects in CDL-based RIS channels

The mobile nature of the users and environmental variations create time-varying characteristics of the channel, which have significant impact on the mmWave communication performance, in practical mobile communication environments. The 3GPP TR 38.901 channel models use the Cluster Delay Line (CDL) channel model with Doppler effects modeled by time varying multipath components for each cluster.

The Doppler frequency shift (DFS) of the r-th propagation ray is defined as:


$$\begin{array}{c} f_{D,r}=\frac{v}{\lambda }\text{cos}(\theta_{v}) \end{array}$$ (21)

Where, λ is the carrier wavelength and $\theta_{v}$ denotes the angle between the direction of motion and the arrival direction of the propagation path.

Thus, the channel coefficient for each ray is now a function of time, and may be written as:


$$\begin{array}{c} h_{r}(t)=a_{r}e^{j\text{2}\pi f_{D,r}t} \end{array}$$ (22)

where $a_{r}$ represents the complex path gain and $f_{D,r}$ denotes the corresponding Doppler frequency.

Doppler spread affects channel coherence time, beamforming stability, and the performance of optimizing the phase of RIS. The larger Doppler spreads result in more channel variations per second and increased channel estimation and reconfiguration frequency for RIS. The coherence time can be estimated as,


$$\begin{array}{c} T_{c}\approx \frac{\text{1}}{f_{D}} \end{array}$$ (23)

Where $f_{D}$ is the highest Doppler frequency that can occur. Hence, the RIS control design for mobile scenarios is gaining importance in high-speed communication scenarios.

## 5. Iterative RIS phase optimization algorithm

The performance of RIS-assisted communication relies heavily on how the phases of the reflective elements are configured. Proper tuning of the phases allows for the coherent combination of the cascaded BS-RIS-UE channel components and therefore maximizes the received signal power. This section formulates the RIS phase optimization problem and provides an efficient iterative solution applicable to CDL-based wideband channels.

### 5.1 Optimization objective

From Section 3, the received signal on *k*^th^ subcarrier is


$$\begin{array}{c} y_{k}={𝐡}_{HU}^{T}{\Phi 𝐇}_{BR}𝐰s_{k}+n_{k} \end{array}$$ (24)

Accordingly, effective channel and received SNR are given as,


$$\begin{array}{c} h_{eff,k}={𝐡}_{RU}^{T}{\Phi 𝐇}_{BR}𝐰 \end{array}$$ (25)

$$\begin{array}{c} \gamma_{k}=\frac{P_{t}{|{\text{h}}_{eff,k}|}^{\text{2}}}{\sigma^{\text{2}}} \end{array}$$ (26)

The RIS phase design aims to maximize received power [30]:


$$\begin{array}{c} \underset{\{\phi_{n}\}}{\text{max}}{|h_{eff,k}|}^{\text{2}}\;s.t.\;\text{0}\leq \phi_{n}<\text{2}\pi \end{array}$$ (27)

This problem is non-convex due to the unit-modulus constraint on RIS elements.

### 5.2 Assumption on channel state information (CSI)

The framework for RIS phase optimization that is considered in this work is based on the assumption of perfect channel state information (CSI) of both BS side and RIS side channels for baseline performance evaluation. It is a common assumption in RIS literature to set an upper bound performance benchmark and to isolate the impact of RIS phase optimization from the channel estimation errors. Optimization of the cascaded channel is thus assumed to be correctly known at the RIS controller. Imperfect CSI is then dealt with by a powerful optimization framework which will be presented later in this section.

### 5.3 Optimal continuous phase solution

Based upon the optimal phase alignment presented in (5), define $v={𝐇}_{BR}𝐰$ and define element-wise cascaded channel components: $g_{n}=h_{RU,n}v_{n}$. To achieve coherent combining, $\phi_{n}^{opt}=-\text{arg}(g_{n})$. Yielding, ${|h}_{eff}|=A\sum_{n=\text{1}}^{N}\left|g_{n}\right|$. This produces the maximum possible coherent gain.

### 5.4 Iterative phase optimization for wideband CDL channels

Because the CDL channel discussed in Section 4 has multiple frequency components, we must ensure that we utilize the correct phase shifts across all OFDM frequencies to maximize the average power received. This means we must solve the following optimization problem,


$$\begin{array}{c} \underset{\phi_{n}}{\text{max}}\sum_{k=\text{1}}^{K}{\left|\sum_{n=\text{1}}^{N}e^{j\phi_{n}}g_{n,k}\right|}^{\text{2}} \end{array}$$ (28)

This can be solved via iterative coordinate descent.

#### 5.4.1 Iterative update rule.

At iteration i, update phase of element n:


$$\begin{array}{c} \phi_{n}^{\left(i+\text{1}\right)}=-arg\left(\sum_{k=\text{1}}^{K}g_{n,k}^{*}\sum_{m\neq n}e^{j\phi_{m}^{(i)}}g_{m,k}\right) \end{array}$$ (29)

The update aligns each element with the aggregate reflected field.

#### 5.4.2 Convergence behaviour.

In the iterative phase alignment algorithm, the phase of each RIS element is optimized iteratively by fixing the others. Each update optimizes the objective function with respect to the RIS element, so that the power received will be non-decreasing after each iteration of the update. As a result, the objective function is monotonic decreasing to a locally optimal solution. For typical RIS configurations with hundreds of reflecting elements (64–256), the simulation results show that convergence usually occurs within 5–15 iterations.

#### 5.4.3 Complexity analysis.

Let N be the number of RIS elements, and K be the number of OFDM subcarriers. The number of calculations per iteration of the RIS elements is evaluated over all the subcarriers, which yields a computational complexity of O(NK) per iteration, where, N= RIS elements, and K = OFDM subcarriers.

Thus, after *I* iterations, the total complexity is O(INK). Compared to SDR-based methods, which are polynomially scalable with RIS size, the proposed iterative algorithm has significantly lower computational complexity and can be implemented in real-time systems with large-scale RISs.

### 5.5 Discrete phase quantization and RIS hardware non-idealities

The RIS phase shifts are assumed to be continuously variable in the above analysis. In practical RIS implementations, however, the phase shifter is a digitally controlled one, and has finite resolution. Thus, each RIS element’s phase response is subject to quantization error, which is limited to a finite number of discrete values, and impacts beamforming accuracy.

For a RIS equipped with b-bit phase shifters, the set of available phase states is given by

Quantized phase values:


$$\begin{array}{c} \phi_{n}\in \left\{\text{0},\frac{\text{2}\pi }{{\text{2}}^{b}},...,\frac{\text{2}\pi ({\text{2}}^{b}-\text{1})}{\pi /{\text{2}}^{b}}\right\} \end{array}$$ (30)

The optimized continuous phase $\phi_{n}$ is quantized to the nearest discrete phase value:

After continuous optimization:


$$\begin{array}{c} \phi_{n}^{quant}=arg\underset{\text{0}\epsilon Q}{\text{min}}|\phi_{n}^{opt}-\theta | \end{array}$$ (31)

The resulting quantization error is expressed as,


$$\begin{array}{c} \epsilon_{n}=\theta -\phi_{n}^{quant} \end{array}$$

The coherent combining efficiency of the reflected signals is decreased by these phase errors, and, in addition, there is a quantization loss in the received power. The degradation in the corresponding beamforming gain can be closely estimated by,


$$\begin{array}{c} \eta_{q}={\left(\frac{\text{sin}\left(\pi /{\text{2}}^{b}\right)}{\pi /{\text{2}}^{b}}\right)}^{\text{2}} \end{array}$$ (32)

However, it has been demonstrated that RIS architectures with 2–6 bit phase shifters can achieve a substantial amount of the beamforming gain and reduce the hardware complexity and implementation cost significantly. So, finite-resolution RIS designs are a good trade-off between performance and practical deployment feasibility.

### 5.6 Robust optimization under channel estimation errors [26]

Though the optimization framework in Section 5.2 assumes perfect CSI, practical RIS deployments are pretentious by channel estimation errors due to mobility, noise, feedback delay, and hardware impairments. These effects can be taken into account by considering the estimated channels as, g^n=gn+ϵn

$g_{n}$ is the true channel and $\epsilon_{n}$ is the estimation error. The RIS phase design problem is then re-formulated as a robust optimization problem to maximize received signal power under channel uncertainty with bounded channel estimation error. A new phase-update rule, which is regularized, is used to ensure robustness to CSI inaccuracies and reduce sensitivity to CSI errors. This powerful formulation allows for a stable RIS operation in real propagation environments and offers greater robustness against channel uncertainty and beam alignment errors. Also, the comparative analysis with different alternative methods are also compared in Table 1 which is given below.

**Table 1:** Comparative analysis with alternative methods.

Method	Complexity	Optimality	Practicality
Semidefinite relaxation	High	Near-optimal	Limited scalability
Genetic algorithms	Very high	Global search	Slow convergence
Deep learning methods	Offline training	Fast inference	Dataset dependent
Iterative alignment (proposed)	Low	Near-optimal	Real-time feasible

Iterative alignment provides balance between performance and computational efficiency.

### 5.7 Beam misalignment and CSI imperfections

The suggested framework is a maximum performance benchmark under perfect beam alignment conditions. In real-world mobile use-cases, beam tracking, aging channels and users moving might cause misaligned beams making it necessary to do research for future improvements in RIS optimizations which take into account user mobility.

## 6. Results and discussion

A comprehensive analysis of the performance of the 6G mmWave communication framework including RIS is also reported for real world propagation scenarios. The simulations used standardized CDL channel models that accurately represent the temporal, spatial, and multipath characteristics of the high frequency wireless system. The analysis considers many performance metrics such as spectral efficiency, SNR, coverage probability, energy efficiency, and localization accuracy. The results show that the RIS is capable of actively controlling the way that electromagnetic waves propagate, thereby providing solutions to the significant path loss and signal blockage problems associated with mmWave frequency bands. The effect of changing the size of the RIS panels, using phased quantized RIS elements, and changing channel types (LOS and NLOS) on link performance was also investigated.

The simulation parameters summarized in Table 2 were used for evaluating the performance of the proposed RIS-assisted communication framework. For simulation, the following parameters were used as summarized in Table 2. The simulations are based on 3GPP TR 38.901 CDL channel model, including both LOS (CDL-E) and NLOS (CDL-D) propagation. Performance metrics are spectral efficiency, received SNR, coverage probability, energy efficiency, localization accuracy and sensing performance.

**Table 2:** Simulation parameters.

*Parameter*	*Value*
Carrier Frequency	28 GHz
Communication Scenario	Downlink BS-RIS-UE
Channel Model	3GPP TR 38.901 CDL
CDL Profiles Considered	CDL-D (NLOS), CDL-E (LOS)
Transmission Scheme	OFDM
Modulation	16-QAM
System Bandwidth	400 MHz
Number of OFDM Subcarriers	1024
Subcarrier Spacing	120 kHz
BS Antenna Configuration	8 × 8 Uniform Planar Array
UE Antenna Configuration	Single Antenna
RIS Element Configurations	16, 32, 64, 128, 200, 256
RIS Reflection Amplitude (A)	0.9
RIS Phase Resolution	Continuous, 2-bit, 3-bit
BS Transmit Power	30 dBm
Noise Figure	7 dB
Receiver Noise Power Density	−174 dBm/Hz
Path-Loss Model	3GPP TR 38.901 mmWave
BS–RIS Distance	100 m
RIS–UE Distance	100-300 m
Propagation Environment	Urban mmWave, LOS/NLOS
User Velocity	3 km/h (pedestrian)
CSI Assumption	Perfect CSI (Baseline)
Optimization Method	Iterative RIS Phase Alignment
Monte Carlo Realizations	1000
Coverage Threshold	5 dB SNR
Localization Metric	RMSE and CRLB
Performance Metrics	Spectral Efficiency, Coverage Probability, SNR, Energy Efficiency, Localization Accuracy

### A) Element scaling and spectral efficiency enhancement

**Fig 2:**
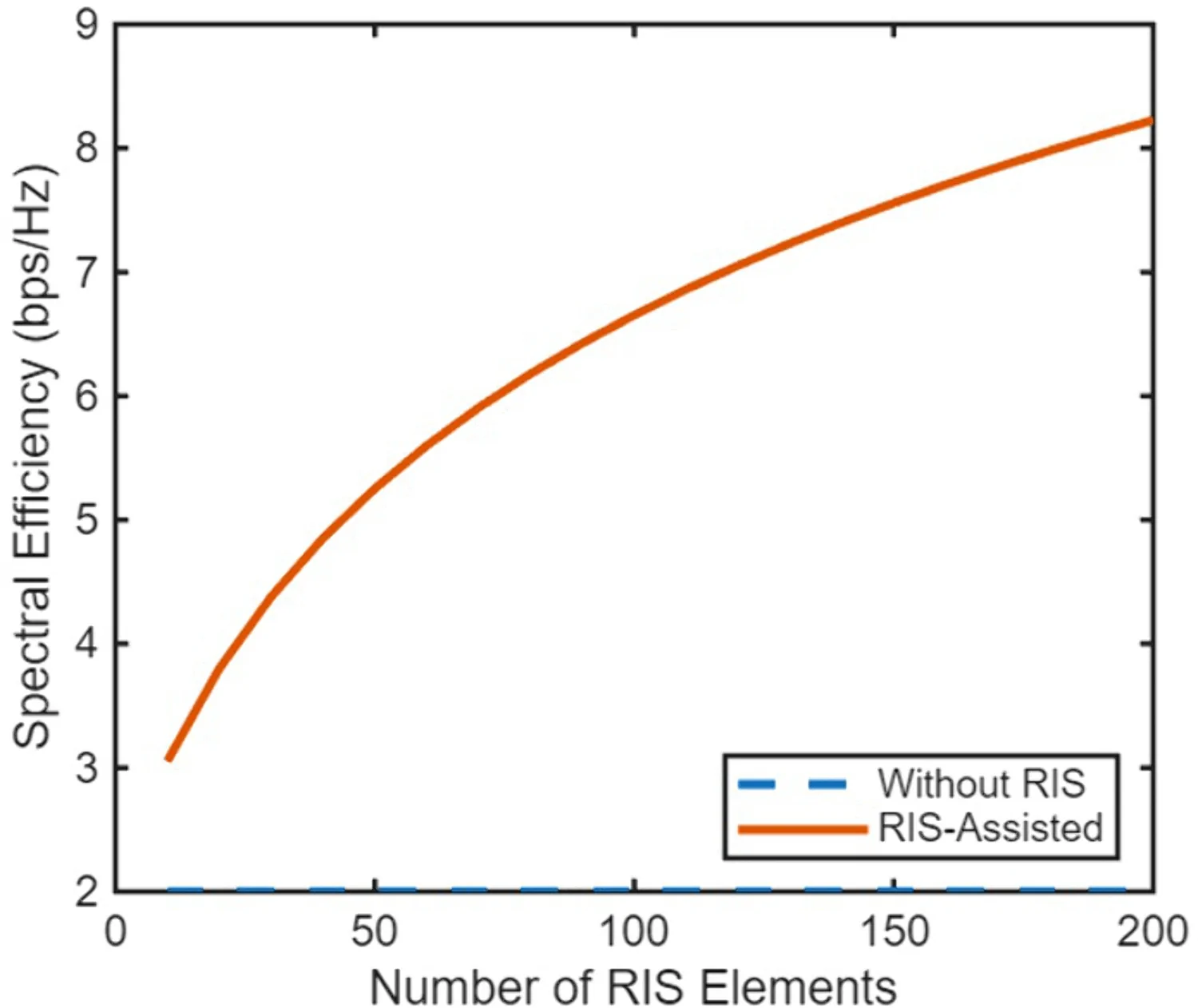
Spectral Efficiency Improvement with RIS Size.

The number of elements deployed at the RIS will inherently determine how well a link using RIS performs. As shown in the figure below (Fig 2), the increase in the number of elements results in an exponential increase in spectral efficiency; therefore, spectral efficiency grows logarithmically with an increase in the element count. The performance of a conventional system (no RIS) would remain relatively stable around 2 bps/Hz as shown below, while the performance of the RIS system would increase substantially (over 8 bps/Hz) when using 200 elements, providing significant benefit to the system at 28 GHz due to the high propagation losses.

The Fig 2 shows the dependency of the achievable spectral efficiency on RIS size. The RIS offers enhanced coherent signal combining to achieve more beamforming gain when the number of reflecting elements grows to 200. The larger the effective aperture is, the more power will be received and therefore the more effective the signal-to-noise ratio (SNR) will be. This ensures that the spectral efficiency achievable by RIS grows monotonously with RIS size. The performance improvement is significant especially in the blockage prone mmWave propagation scenario where RIS-assisted reflections mitigate severe path loss and limited scattering.

The coverage enhancement results show the possibility of RIS technology in extending the coverage area beyond the line-of-sight region. As the number of RIS elements increases, the reflected signal strength is also increased and the coverage holes due to blockage and shadowing are reduced. Larger RIS configurations provide for much higher coverage probability due to the ability of the reflected beam to be focused to the desired user. The observations validate that RIS deployment is an effective solution to enhance coverage reliability in dense urban and indoor mmWave environments.

**Fig 3:**
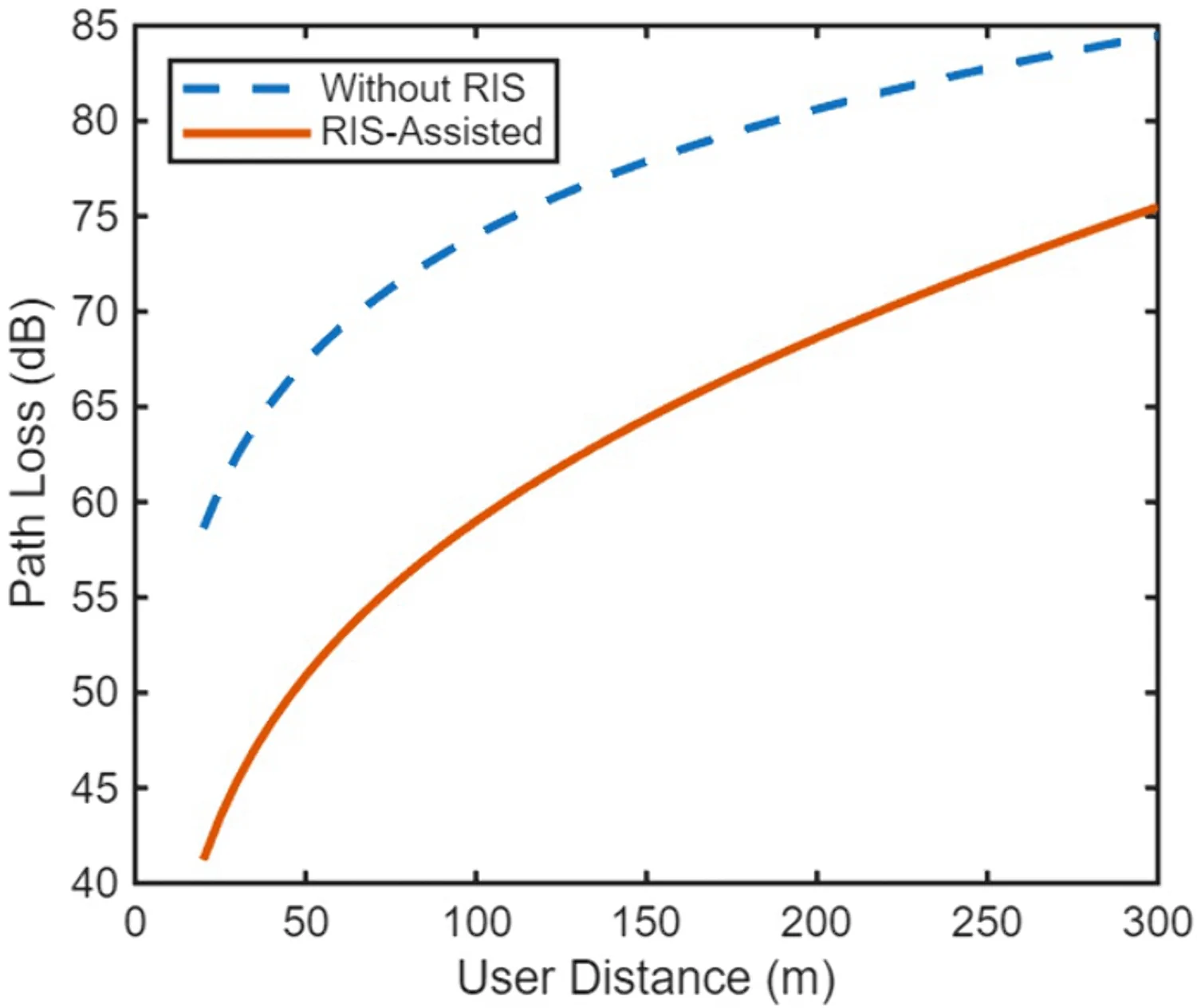
Coverage Improvement with RIS Assistance.

As shown in Fig 3, the improvement shown is due to changes in the gain produced by the array as a function of the number of antennas. When comparing the gains obtained by increasing the number of antennas from 16 to 256, we see that the theoretical prediction of N² increase in power associated with passive beamforming is confirmed by an increase from about 24 dB to 48 dB gain.

### B) Influence of channel profiles and signal quality

**Fig 4:**
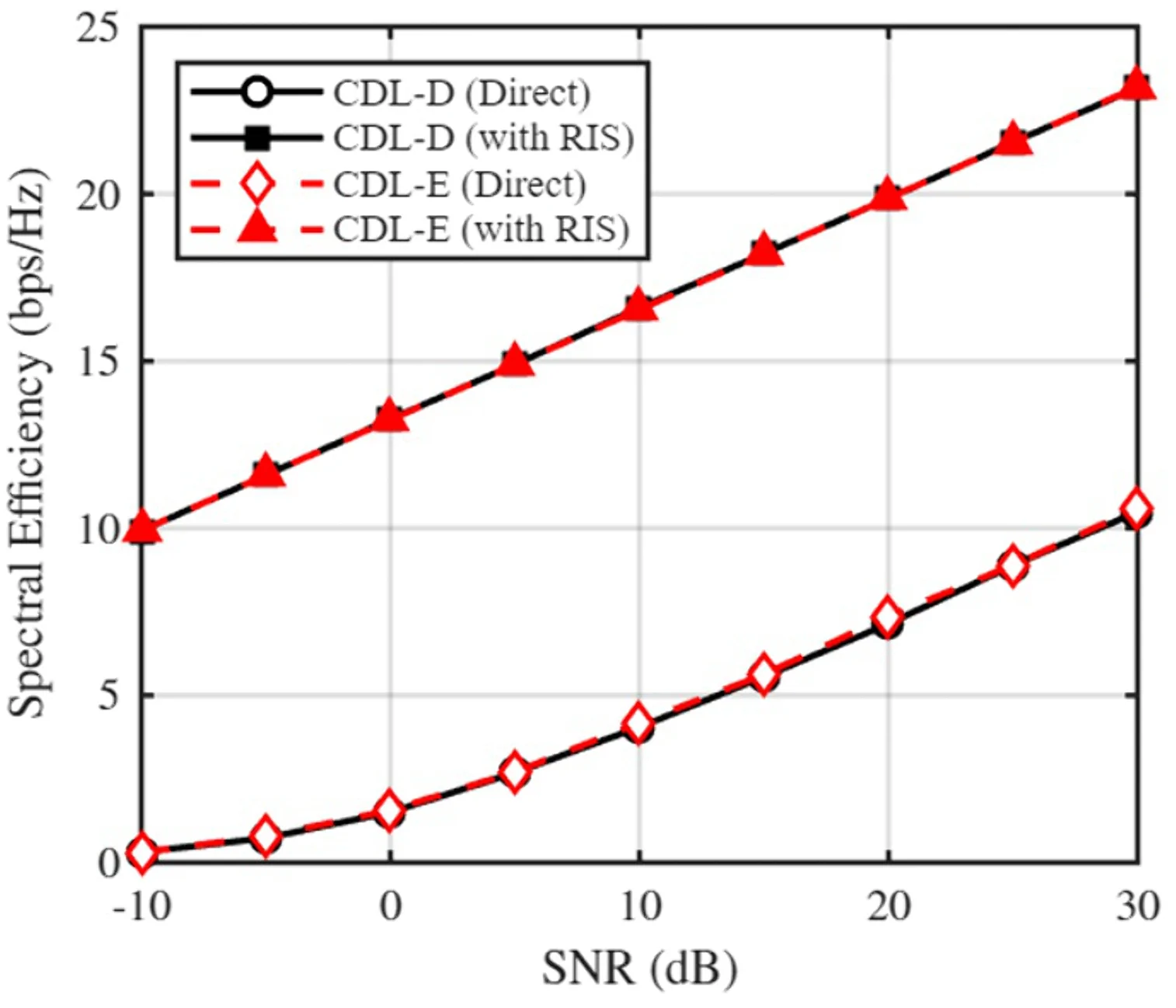
Performance Comparison: CDL-D vs CDL-E.

**Fig 5:**
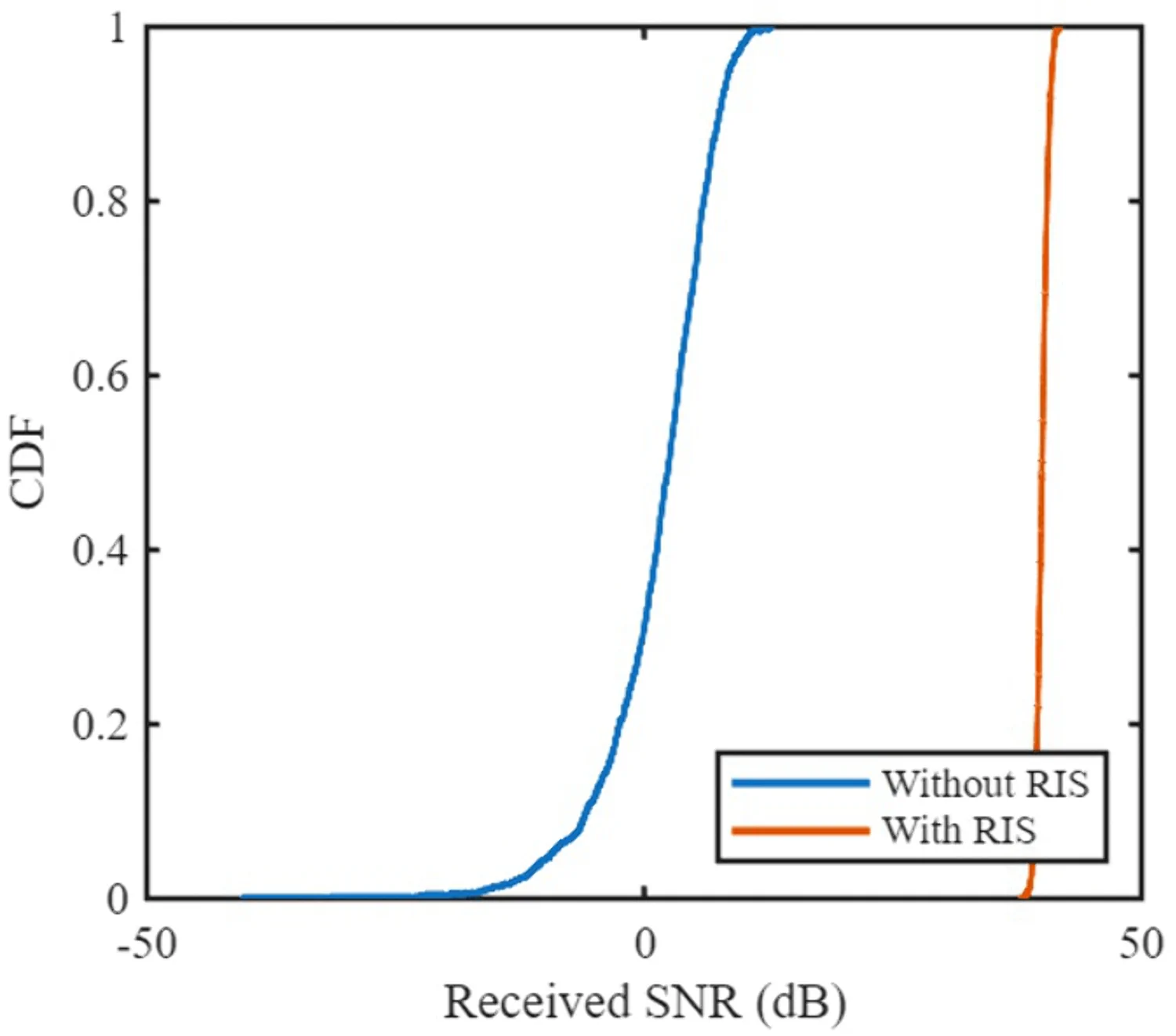
CDF of Received SNR under CDL-D Channel.

The system performance was explored for two separate propagation scenarios from the 3GPP TR 38.901 specifications of CDL-D, which simulates an NLOS condition, and CDL-E, which simulates an LOS situation. The graph in Fig 4 demonstrates that both scenarios produce significant increases in performance. In the CDL-D case, where there is a substantial obstruction of the direct path of transmission, the RIS enables the development of an alternate way of propagating to emulate an LOS scenario and increases spectral efficiency from close to 0 bps/Hz to more than 23 bps/Hz at high SNR conditions. The statistical distribution of the received SNR is presented in Fig 5 with empirical cumulative distribution function (CDF) curves. The unassisted system has a broad spread of SNRs with most values very close to 0 dB; however, the RIS-assisted link has shifted the complete distribution to the right, with all values clustering around 40 dB which shows that a stable and reliable wireless connection is likely.

### C) Coverage extension and path loss reduction

**Fig 6:**
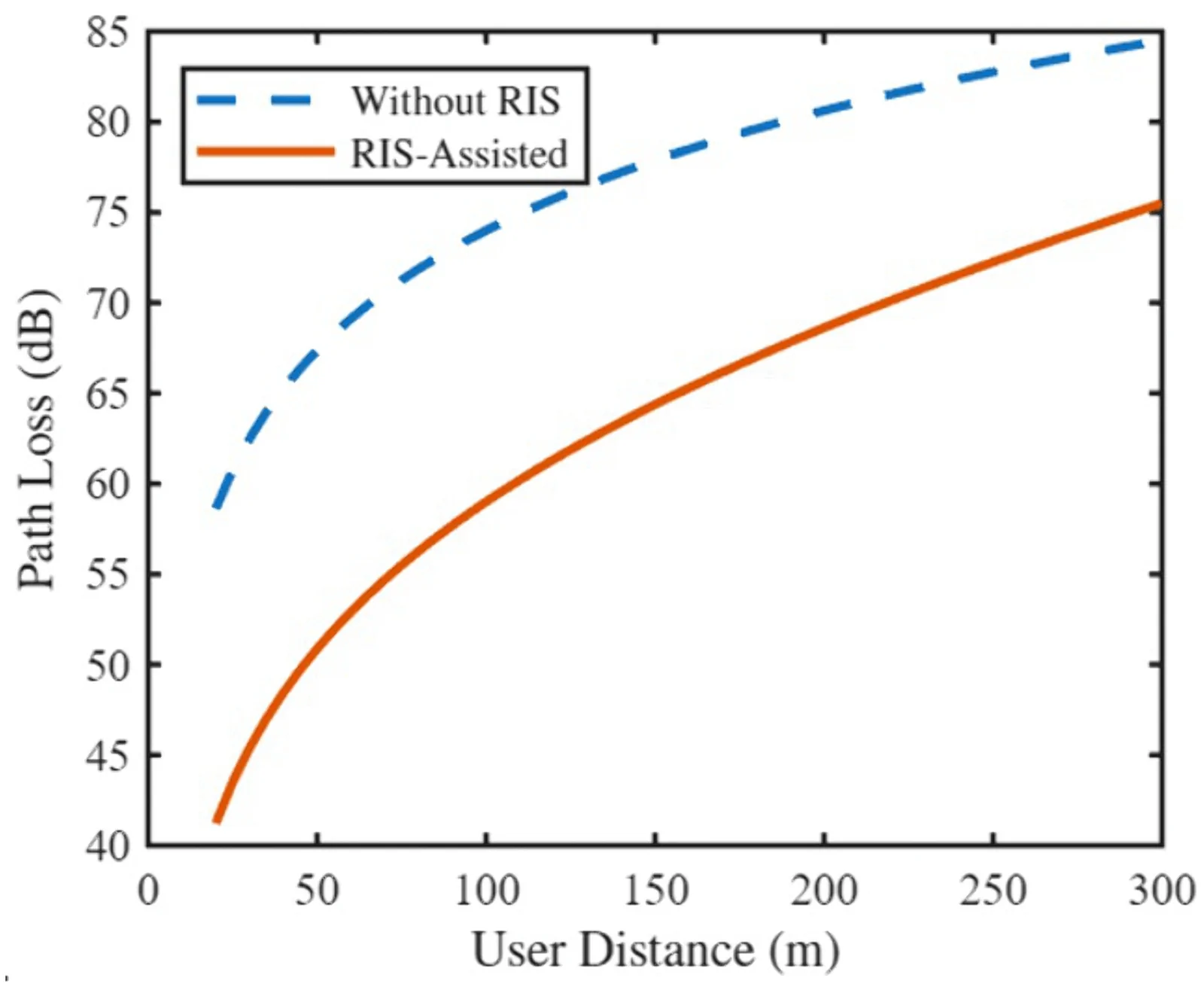
Coverage Improvement with RIS Assistance.

The continued challenge of rapid signal attenuation at distance remains one of the most common challenges in mmWave deployments. Path loss for every meters of user separation distance can be seen in Fig 6, which shows the amount of signal loss would vary from 10 to 15 dB in comparison to a direct path over 300-meters of distance. This shows that reflective beamforming is very effective over this distance, where a consistent advantage remains between the two different paths.

**Fig 7:**
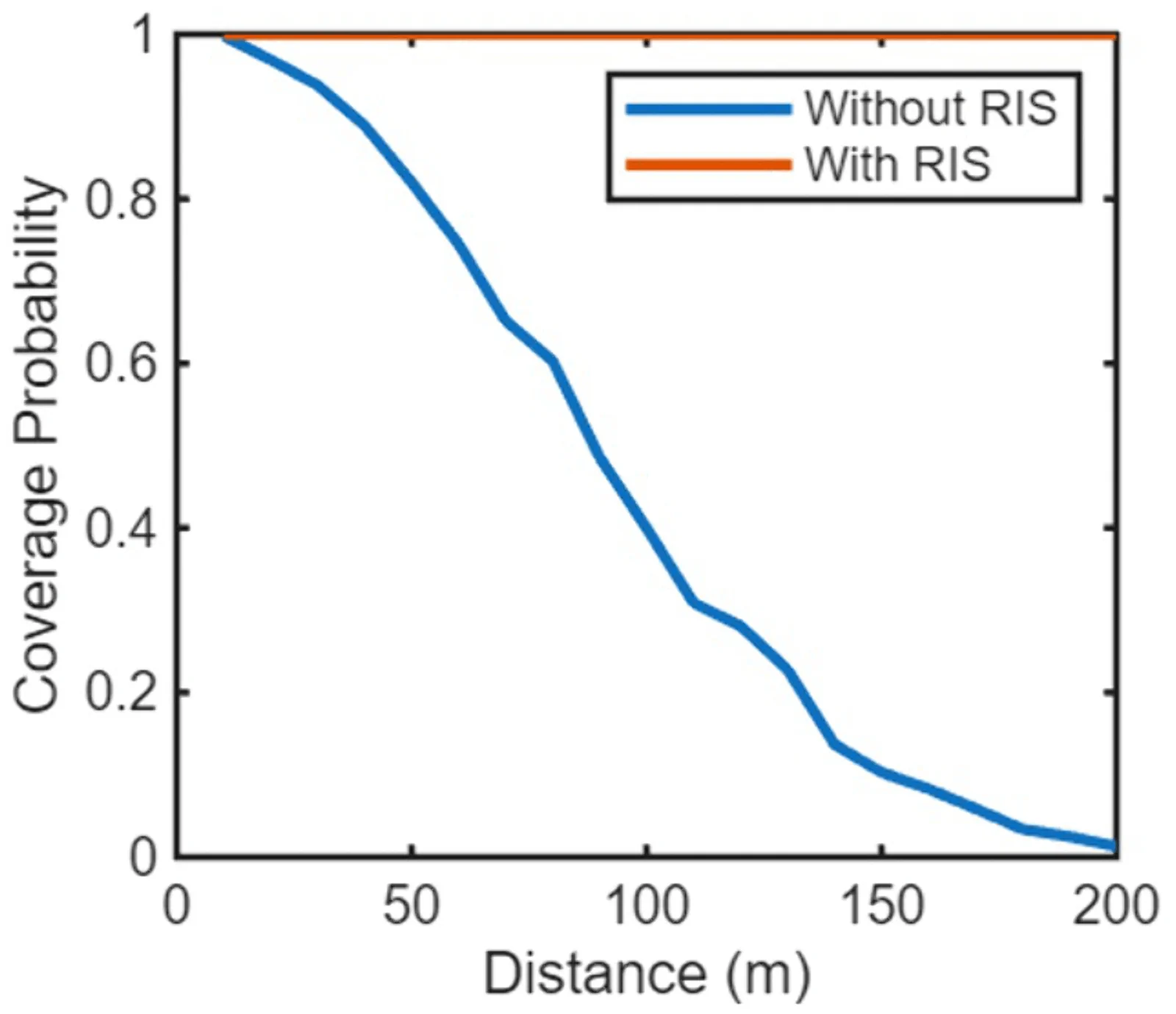
Coverage probability improvement with RIS.

When looking at continuity of service as described by Fig 7, it shows that there would be essentially no probability of maintaining a signal with a minimum reliability level of 5 dB for distances greater than 200 meters without the use of an RIS; however, with the use of an RIS, that same probability would remain one over the entire distance tested. This further illustrates that with an RIS, even in locations that have been affected by shadowing or obstructed by other materials with a low signal-to-noise ratio, signal strength will remain at or above the minimum required to maintain connectivity.

### D ) Practical hardware constraints and power efficiency

The measured energy-efficiency improvements are despite taking account of the power used by the RIS controller and the phase control power. The RIS elements work without any RF amplification or regeneration unlike active relays, they have significantly lower operation power requirements. Likewise, the reflectivity of the RIS adds extra reflected paths and enhances the angular resolution and multipath diversity, thus boosting the localization performance. Thus, the RIS-assisted system can obtain the smaller positioning error and better sensing capability than the conventional communication system without RIS assistance.

The impact of hardware limitations on RIS has to be taken into consideration for real-world deployment of RIS. The phase shifters’ finite resolution and the power consumption of the controller are two important factors in the success of RIS. In evaluating real-world deployment, it is essential to understand how much degradation there is in spectral efficiency when you operate your RIS with either 2 or 3 bit phase control.

**Fig 8:**
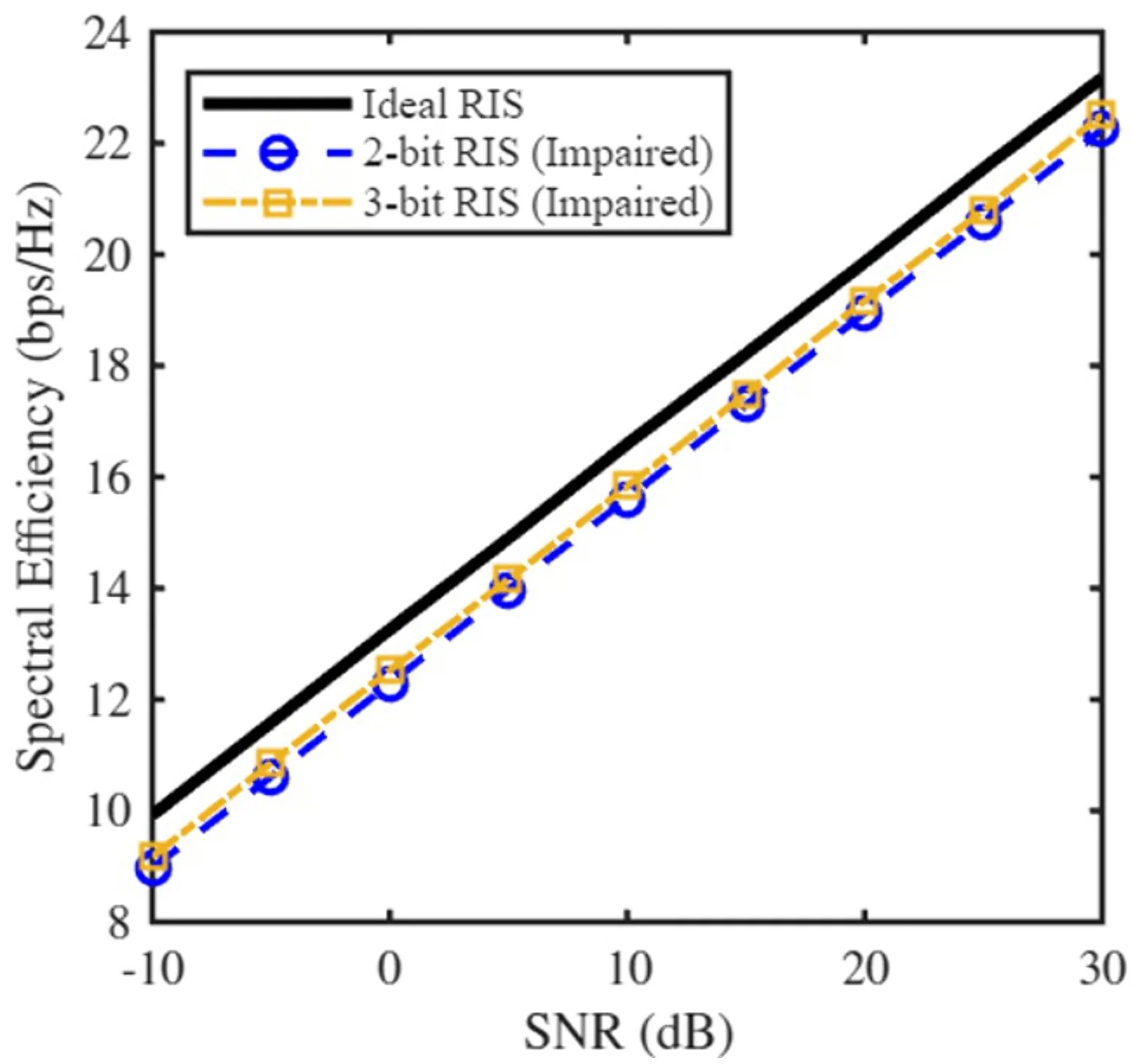
The impact of hardware limitations on RIS.

The results plotted on Fig 8 show that spectral efficiency will degrade considerably by using low resolution (e.g., 2 or 3 bit) phase control; however, the 3-bit quantized phase system still performs very closely to the theoretical continuous phase system. This leads to an important conclusion that cost effective, low-complexity hardware can be used effectively in real-world 6G implementations and provide useful/meaningful performance.

**Fig 9:**
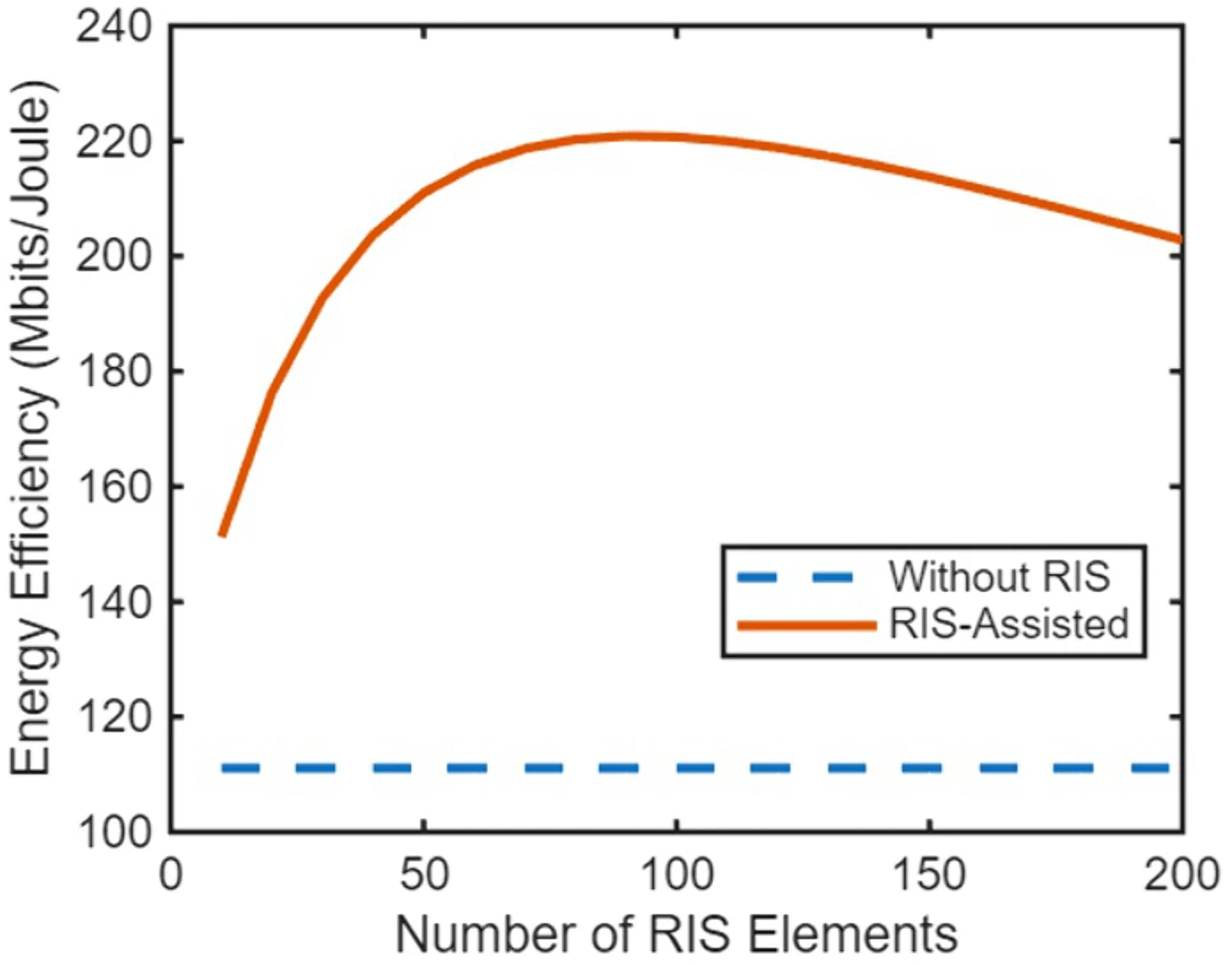
Energy Efficiency Improvement with RIS Size.

Additionally, we examine the energy efficiency of both systems by comparing their energy efficiency from a power consumption perspective according to Fig 9. Although the RIS controller adds additional overhead, the large improvement in spectral efficiency yields a net gain in bits-per-joule (i.e., approximately 5 bits/J for the RIS-assisted link vs. approximately 3 bits/J for the conventional baseline link).

The total system power consumption in the energy-efficiency assessment also accounts for the power of the RIS controller, the power of the phase-control circuitry, and the overhead for signal processing due to the RIS configuration. These extra parts add to the overall energy use, but the high throughput improvements brought by RIS-assisted beamforming more than offsets the overhead. Thus, the RIS-assisted system can still achieve better energy efficiency than traditional communication links and active relay systems.

### E ) Localization accuracy enhancement

**Fig 10:**
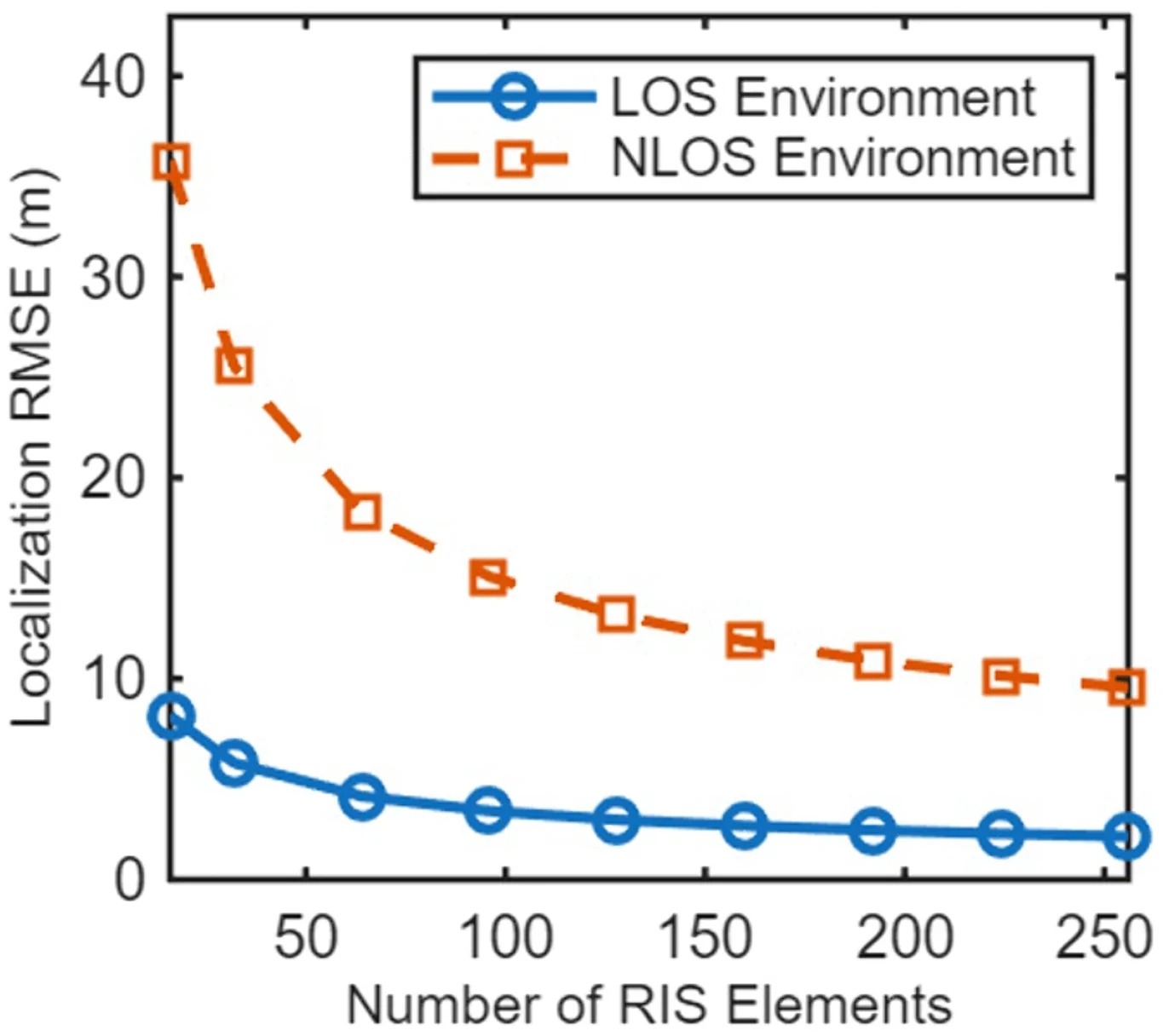
Localization Accuracy Improvement using RIS: LOS vs NLOS Urban Channel Conditions.

Another important aspect of RIS that isn’t typically recognized is its ability to enable high-accuracy spatial positioning, which has significant implications for resilient navigation and intelligent infrastructure. As illustrated in Fig 10, localization RMSE is plotted against the number of elements. In nonline-of-sight (NLOS) scenarios, RMSE is reduced from about 35 meters to under 10 meters when the number of elements reaches 250, which represents a large improvement in accuracy.

The localization results are computed using FIM/CRLB framework discussed in Section 3. The AoA and delay vector obtained from the propagation channel assisted by RIS are used to estimate the user position. The more RIS elements the reflected wavefront has, the more direction it will have, which will lead to better angular resolution and less uncertainty in the estimation. Therefore, higher RIS apertures result in the better localization accuracy with lower CRLB values.

**Fig 11:**
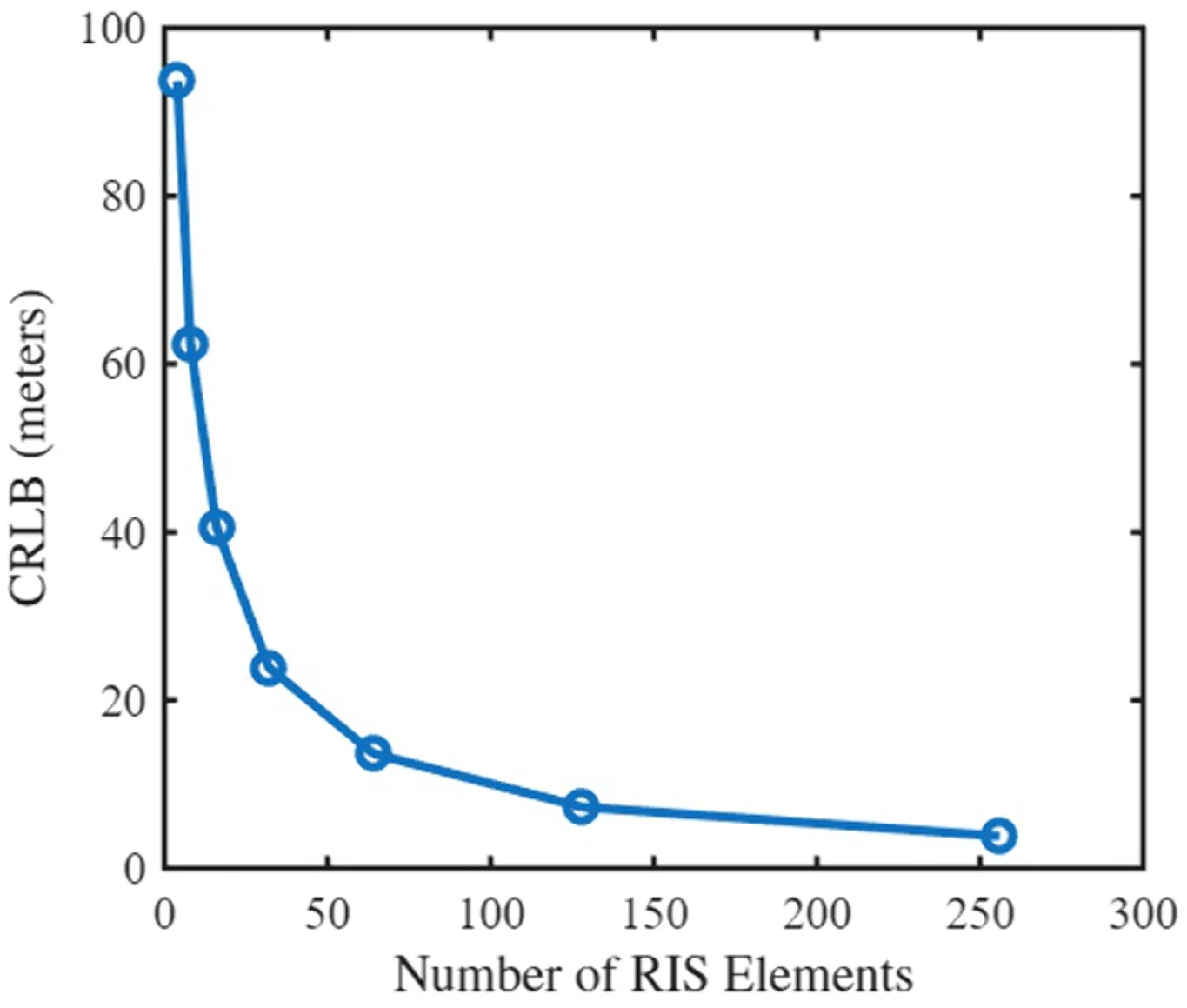
CRLB vs RIS Elements.

To further support this observation, the theoretical performance bounds (CRLB) are shown in Fig 11. The CRLB is sharply reduced as the number of elements increases; therefore, when the number of elements approaches 250, there is an achievable positioning accuracy of less than 5 metres, demonstrating that RIS can be used as a dual-purpose physical layer to provide both reliable communication and accurate user positioning in local urban deployments of smart cities.

#### Impact of mobility and doppler effects.

CDL model can synthesise Doppler spectra and time-varying channel coefficients, which can help evaluate the RIS-assisted communication performance under user mobility. The more the channels are Doppler spread, the more the coherence time will shorten; this can lead to a greater number of phase updates for the RIS. However, the proposed optimization framework is still viable for moderate mobility conditions, which are commonly seen in urban mmWave deployments.

**Fig 12:**
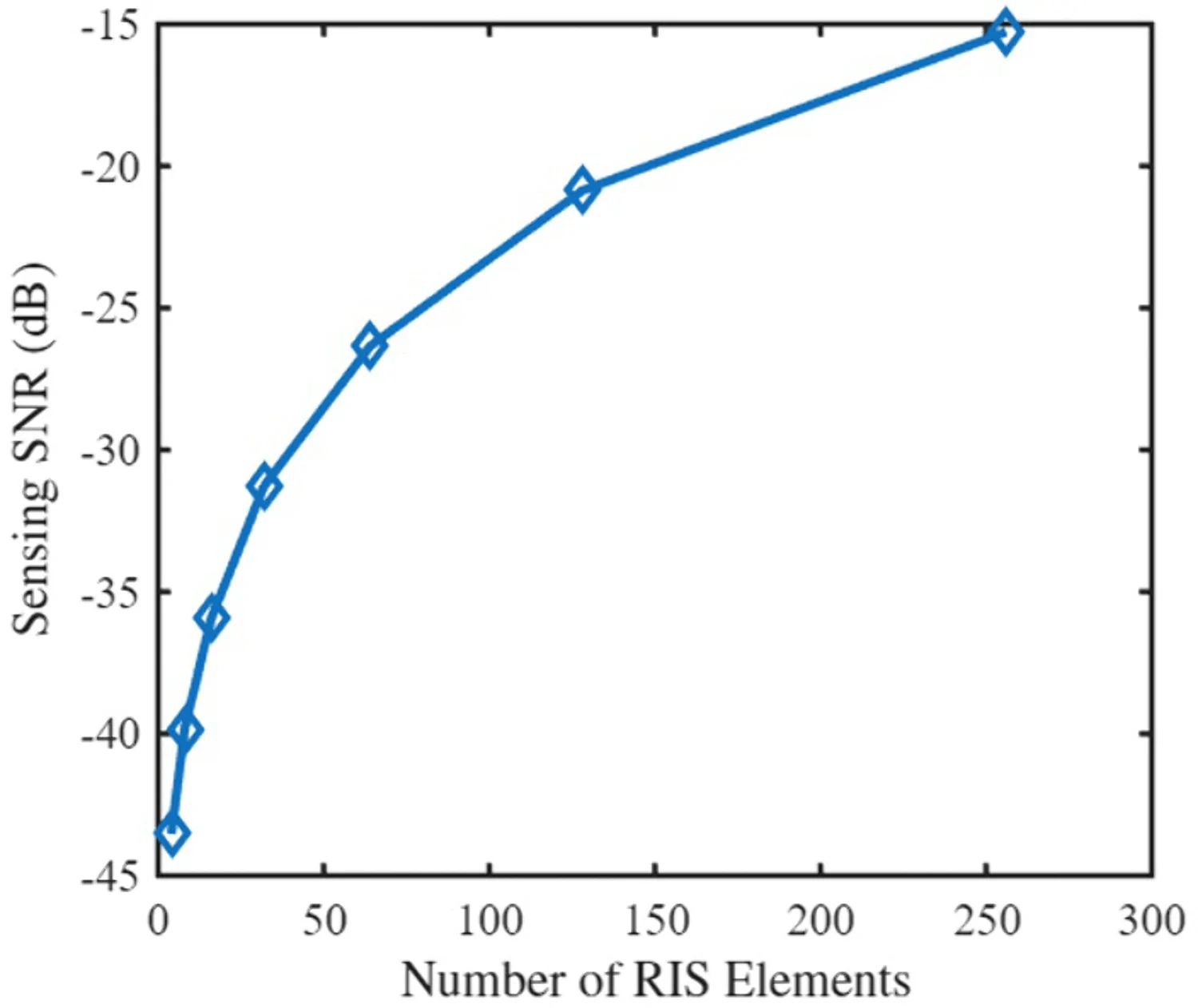
Joint Communication and Sensing Performance.

The reformulated sensing performance of the system, which relies on an RIS to assist in sensing, is evaluated as a function of the number of passive reflective elements. The measured SNR values are displayed in Fig 12. If look carefully at the SNR plot, the number of reflecting elements increases, the SNR of the sensing improves, but not consistently. When the reflecting element array is small, the system does not have enough signal energy to redirect towards the target object, resulting in an SNR of approximately −43 dB. This is understandable; A small aperture will limit the spatial resolution and directional control of the reflected wavefront. As more reflective elements are added to the reflector array, the additional reflected paths will accumulate coherently in the receiver, which will produce an increase in the received signal strength.

Noticing the most pronounced gains arises from improvements in lower end of the element range (N = 10–60) where SNR climbs terrifically. After this break-point the curve starts to flatten out and any further addition to the array will yield only small increase (incremental gain) improvements in SNR over previous number of elements. By the time we reach an element size of 256, it will obtain an SNR value close to -15dB indicating an overall great increase from the initial value but there will be minimal incremental value added from the addition of each new element at this large size.

This trend has practical considerations for system designers. Generally speaking, the initial steep rise in the SNR curve suggests that even a relatively small RIS will significantly improve sensing performance compared with a system that uses no surface enhancement. On the other hand, the flatter nature of the SNR curve beyond some threshold indicates that there is no practical or economic benefit to growing the array of elements indefinitely. Therefore, for practical implementation, we recommend an element configuration that balances cost of supporting hardware versus achievable SNR.

RIS has demonstrated quantifiable improvements in communication reliability, signal coverage, energy efficiency and spatial location. Future enhancements of RIS are expected to be a base technology for developing next generation wireless intelligent networks.

## 7. Conclusion

In this paper, developed a model-based analysis of RIS-assisted beamforming to investigate its ability to improve the performance of mmWave links in future 6G networks by means of a realistic CDL-based channel modeling framework. Built a cascaded BS-RIS-UE system that utilized OFDM transmissions and adaptively controlled the phases of the RIS elements and investigated the behavior of the signals at mmWave frequencies in environments with substantial blockage. An iterative optimization method was used to achieve reliable coherent signal combining on RIS elements with minimal computational costs. The simulation results demonstrated that RIS-assisted systems exceeded the performance of traditional direct link systems in all assessed categories especially with no direct line of sight. The relationship between the number of RIS elements and the amount of array gain produced was confirmed, and it was determined that using low-resolution phase controls on the RIS elements would produce sufficiently near ideal performance in practice thus establishing the feasibility of deploying RIS in real-world environments. Further, confirmed that RIS can enhance localization accuracy by providing increased spatial diversity and angular resolution. Collectively, these results provide compelling evidence that RIS has great potential for enabling wireless infrastructures for 6G that are highly reliable, energy efficient and capable of providing sensing services. Future research will include developing optimization methodologies for RIS that provide wideband transmission, creating accurate channel models for electrically large surfaces operating in the near-field, as well as machine learning methods which enable contextually aware and dynamically adjusting configurations of RIS in rapidly changing propagation environments.
